# Use of halo fixation therapy for traumatic cranio-cervical instability in children: a systematic review

**DOI:** 10.1007/s00068-021-01849-z

**Published:** 2021-12-09

**Authors:** Mohammed Banat, Martin Vychopen, Johannes Wach, Abdallah Salemdawod, Jasmin Scorzin, Hartmut Vatter

**Affiliations:** Department of Neurosurgery, University of Bonn, Venusberg-Campus 1, Building 81, 53127 Bonn, Germany

**Keywords:** Traumatic cranio-cervical instability, Pediatric, Halo fixation, Spine surgery

## Abstract

**Purpose:**

Traumatic cranio-cervical instability in childhood is rare and constitutes a challenge for the treating surgeon. The aim of therapy is to restore cervical stability without limiting the range of motion. The goal of this systematic review was to find out whether, over the last 10 years, halo fixation (HF) could still be considered a successful treatment option without major risks or complications.

**Methods:**

We analyzed studies describing the use of HF in traumatic injuries of the cranio-cervical junction in children under the age of 17. Searches were performed in PubMed, MEDLINE and Embase databases for the years from 2010 to 2020. The general success rate, the success rate related to underlying pathologies, and complication rates were evaluated.

**Results:**

The main indications for HF range from pre-surgical correction to postoperative fusion support. C2 is the most frequently injured vertebra in children. The overall success rate of HF was very high. Evaluation according to the underlying pathology showed that, except for atlanto-occipital dislocation, HF generates high fusion rates among different patient cohorts, mainly in C2 vertebra injuries and atlantoaxial rotatory subluxation. Only minor complications were reported, such as pin infections.

**Conclusion:**

The current data show that, when used according to the appropriate indication, HF is an effective conservative treatment option for cranio-cervical instability, associated with only minor complications.

## Introduction

The use of halo fixation (HF) to treat cranio-cervical instability was first described in 1968 [32]. HF is in widespread use to treat a variety of disorders and pathologies in children and adults [4, 7, 25, 33, 51]. The main indications for HF range from pre-surgical correction [8, 19, 28, 43] to postoperative fusion support [18, 21]. Used as an alternative to surgical treatment, HF enables the treating physician to preserve neck mobility and avoid side effects, such as restricted neck mobility and interference with spinal growth potential [8, 9, 28].

Traumatic cervical spine injuries in children are rare. Deciding whether to use HF to treat traumatic injuries of the upper cervical spine in children is challenging. The literature only records individual cases [36, 52] and series [12, 20, 40]. There are no standard recommendations for its pre-surgical or postoperative use, nor for its use as a conservative therapy option [3, 15, 27] or in combination with surgery [41, 51]. The decision to use HF has to consider possible complications as well as indications and the likelihood of success [9, 14, 23, 26, 34, 49].

The goal of this systematic review was to find out whether, over the last 10 years, HF could still be considered a successful treatment option without major risks or complications.

## Methods

In this systemic review, we analyzed the success rates of HF for traumatic cervical instability in children. We searched the PubMed, MEDLINE and Embase databases for articles published between 2010 and 2020, using the terms “halo fixation”, “cranio-cervical instability”, and “cervical spine injury in children”. 488 papers were identified (after removal of duplicates). We included all clinical publications describing the use of HF as the primary therapy in traumatic injuries of the cranio-cervical junction. We excluded studies where HF was used for non-traumatic pathologies and for patients over the age of 17.

**Fig. 1:**
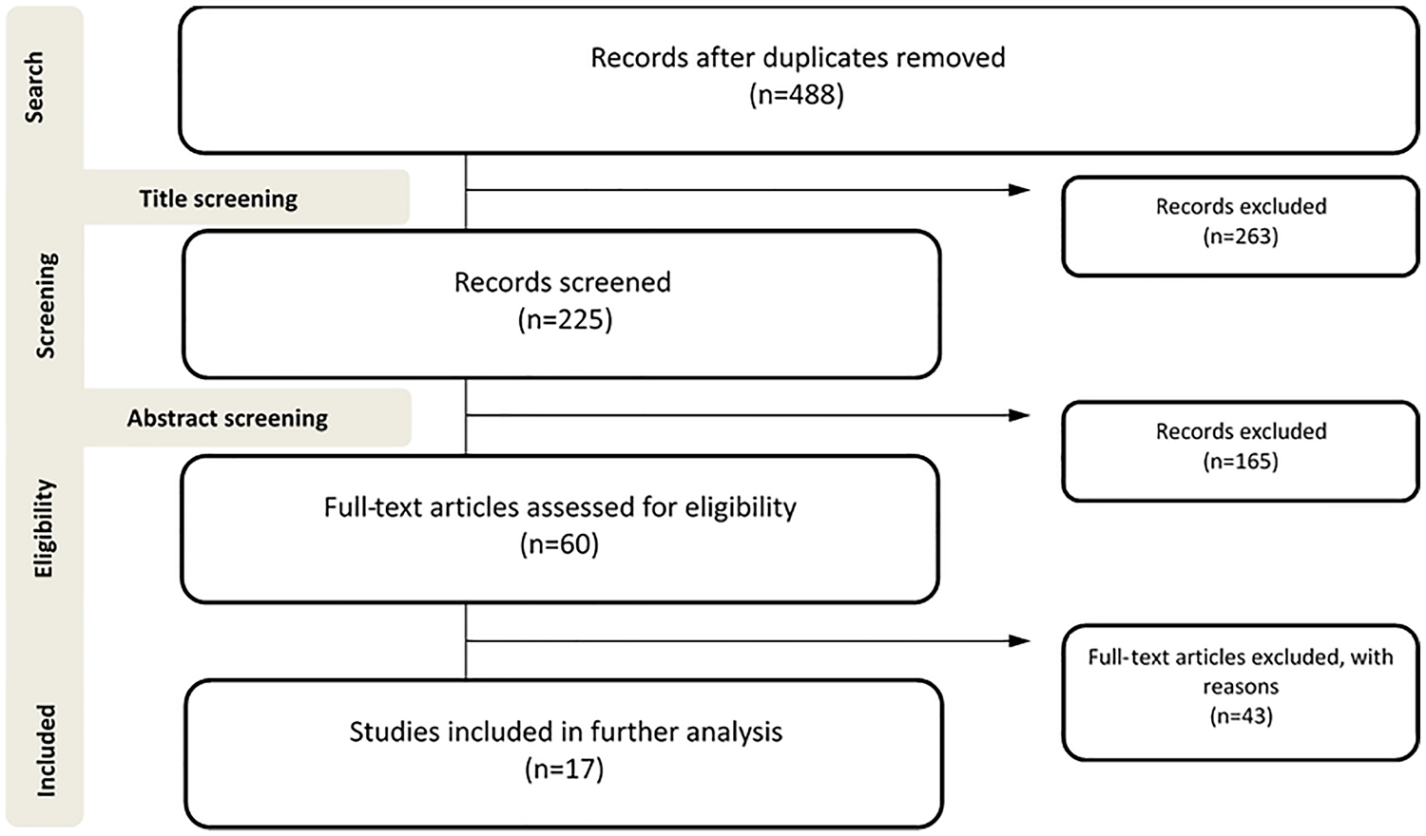
Chart showing the search strategy

263 papers were excluded after title screening, and 165 were excluded after reviewing the abstract. After analysis of the full text, 41 further studies were excluded, resulting in 17 publications that fulfilled the inclusion criteria (Fig. 1)

.

The patient cohorts were stratified according to the most common underlying pathologies, namely atlanto-occipital dislocation (AOD), atlantoaxial rotatory subluxation (AARS), occipital condyle fracture (OCF), and traumatic injuries of the C2 vertebra.

## Results

### Summary of studies

Table 1 shows summary data from all included studies*.*

**Table 1:** Studies included

Study	Underlying pathology	Number of patients treated with HF	Treatment aim	Number of surgical conversions	Complications
Vachta 2020 ^[48]^	AOD	4	Stabilization	1 secondary instrumentation	4 deaths
Abel T 2020 ^[1]^	AOD	8	No fusion, neurologically intact, or stable	NA	Skin irritation
Powell 2017 ^[35]^	AARS vs. CSI	9			
	Immobilization	NA	NA		
Glotzbecker 2014 ^[17]^	AARS	3	Fusion	NA	Neck pain, rotation reduced
Bakshi 2015 ^[6]^	AARS + OCF	11 AARS 2 OCF	Stability	NA	Skin irritation
Verhofste 2019 ^[50]^	AARS	7	Fusion and angle correction	NA	Pin infection
Chechik 2013 ^[11]^	AARS	1	Reduction of rotation and improve of motion	NA	NA
Misfud 2016 ^[29]^	AARS	1	Fusion	NA	Pin infections
Tauchi 2011 ^[44]^	AARS	2	Fusion and deformity correction	NA	NA
Tomaszewski 2018 ^[45]^	C2 fracture	6	Fusion	NA	NA
Rusin 2015 ^[37]^	C2 fracture	6	Fusion and correction of displaced and angulated fractures	NA	NA
Shammassian 2016 ^[39]^	C2 fracture	1	Fusion	NA	NA
Tomaszewski 2014 ^[47]^	Upper cervical spine fracture	6	Fusion	NA	Pin infection
Stulík 2013 ^[42]^	Upper cervical spine fracture	4	Bone healing	NA	NA
Mitchell 2019 ^[30]^	Upper cervical spine fracture	6 patients with HF in addition to surgery (after)	Fusion	NA	Pin infection
Hassler 2020 ^[18]^	Injury of cranio-cervical junction	4 patients with HF in addition to surgery (after)	Fusion	NA	NA
Takahashi 2016 ^[43]^	C1-2 fracture	1 patient with HF in addition to surgery (before)	NA	NA	NA

### Atlanto-occipital dislocation (AOD)

We found two studies about HF therapy for AOD. Vachata et al. [48] reported that “two patients were in severe neurological states, with lesions of the upper cervical spinal cord and medulla oblongata on MRI, these patients were treated with external HF and died within 3 days of the trauma” [48]. In contrast, Able et al. recently reported that treatment with HF was a safe, viable, and definitive treatment option for selected children with AOD [1]. Although several factors biased their results, Able et al. reported good outcomes in eight out of ten patients with AOD (aged between newborn and 17) whose only therapy was external fixation with HF. The treatment lasted 3 months and only a pin-site complication was the only reported complication was pin-site complication [1].

### Atlantoaxial rotatory subluxation (AARS)

We identified seven publications presenting conservative treatment with HF in patients with traumatic AARS. The results and success rates are presented in Table 2 [6, 11, 17, 29, 35, 44, 50].

**Table 2:** Summary of studies with AARS

Study	HF treatment	Progression of subluxation	Surgically fused	HF successful	
Powell et al. ^[35]^	9	NA	NA		9
Glotzbecker et al. ^[17]^	3 primary	NA	NA		3
	1 secondary				NA
Bakhshi et al. ^[6]^	11	NA	NA		11
Verhofste et al. ^[50]^	7	NA	NA		7
Chechik et al. ^[11]^	1	NA	NA		1
Misfud et al. ^[29]^	1	NA	NA		1
Tauchi et al. ^[44]^	2	NA	NA		2

Bakhshi et al. used primary pinless HF in 11 patients with an average age of 6.04 years. They reported success in every case [6].

Tauchi el al. treated seven children with HF. Six of these demonstrated remodeling of the deformed vertebra. Only one patient showed greater deformity and needed surgical C1-2 fixation [44]. Powel et al. [35] retrospectively describe a higher rate of traction in nine patients treated with HF (mean age 7.7 years). Two of these received secondary surgical fusion due to HF failure. Glotzbecker et al. reported on three patients with chronic traumatic AARS (average age of 7.2 years) who received primary conservative HF and another child who received secondary HF [17]. The only complication was mild neck pain and reduction of the cervical range of motion. Verhofste et al. reported that three out of seven patients successfully received primary HF and the other four received it in addition to surgery. Their mean age was 11.3 ± 5.58 years [50]. Chechik et al. successfully treated one girl, aged 7 [11]. Mifsud et al. reported on successful HF therapy for AARS [29].

### Occipital condylar fracture (OCF)

Two patients with OCF were successfully treated with HF [6]. Mean therapy duration was 51 days. Skin irritation was the only reported complication. The average age was 12.61 years.

### Traumatic injury of C2 vertebra

We identified eight studies, five of them favoring primary therapy with HF [37, 39, 42, 46, 47]. Takahashi et al. [43] used HF prior to surgery and other authors used it after surgery [18, 30]. The principal purpose of HF therapy for traumatic C2 fracture with instability is fusion or bone healing [39, 42, 46, 47].

- Odontoid fracture—predominant injury in pediatric patients
  C2 is the most commonly injured cervical vertebra in children [27]. This is due to the instability of the unfused odontoid synchondrosis (the final fusion happens between 5 and 7 years of age) [38]. The treatment of choice is HF with close radiological monitoring [16]. This enables the fracture to heal on its own, thus sparing the patient the limitation in movement reported after surgical fusion. The Neck Disability Index (NDI) for patients undergoing surgery because of C2 fracture can reach up to 44.4% [46], making HF the therapy of choice that prevents possible movement restrictions.
  Surgical fixation is indicated for patients where HF failed to achieve fusion. Fulkerson et al. recommend that early surgical intervention should be considered in cases of odontoid angulation (> 30°), significant displacement (> 11%), and symptoms of spinal cord injury due to a higher probability of ligament disruption and greater instability of the fracture [16].
- Synchondrosal fracture—new classification system
  A new classification of C2 synchondrosal fractures in children has been proposed by Rusin et al. [37]. Type I fractures, described in 64% of the cases, were only treated with primary surgical fixation in two cases and with primary HF in six cases. HF failed to achieve fusion in one patient and secondary surgical fixation was necessary. Type II fractures are rare, described in only two cases, and the therapy of choice was HF.
  Primary surgical stabilization was mainly indicated for children with major displacement (subtype III). Subtype d fracture displacement was fatal in both cases [37]
- Hangman’s fracture—C2 injury

A hangman’s fracture is a hyperextension C2 injury, which is almost exclusively seen in children under 2 years of age. The incidence increases again from the age of 16, and then has the same characteristics as C2 fractures in adults [38, 42]. The recommended treatment for a hangman’s fracture varies according to the type and severity of the fracture instability. Types I and II are suitable for conservative treatment with HF, whereas patients with type IIA and III should undergo surgical instrumentation. However, internal fixation has a higher probability of secondary deformities and limitation in growth potential. HF seems to be beneficial not only in preserving movement capacity, but also in avoiding late consequences of surgery [31].

## Discussion

### Atlanto-occipital dislocation (AOD)

AOD is a devastating injury with high mortality, usually caused by high energy trauma [5, 40, 48]. Traction should be avoided because of the risk of deterioration [45]. The definitive treatment of traumatic AOD remains controversial, but a widely held view is that all patients with AOD should be treated with early surgical fusion (internal dorsal fixation and arthrodesis) [5, 45, 48]. In contrast to this opinion, external immobilization with HF on a small group of eight patients was found to be viable, safe and effective [1].

### Atlantoaxial rotatory subluxation (AARS)

AARS is one of the more common cervical injuries which, due to their ligament elasticity, is found almost exclusively in children [35]. The decision of this study to prefer surgery to HF was based on the time from symptom onset, although very good outcomes have been described for delayed HF therapy in combination with traction [6, 11, 17, 24, 29, 35, 44, 50] or for delayed therapy with pinless HF [6]. The decision to prefer surgery to HF was based on the time from symptom onset. Although very good outcomes have been described after delayed therapy with HF [19, 29, 52], chronic pain lasting more than 6 weeks or acute neurological deficits are the most common indications for primary surgical therapy.

The main recommendation in the literature for this injury type is HF therapy.

### Occipital condyle fracture (OCF)

OCF is a rare cervical spine injury after blunt trauma with a prevalence of 1.4–4.4% [3]. Generally, non-surgical management is recommended [6]. The Anderson–Montesano classification divides OCF into 3 types and non-surgical management with cervical orthosis is recommended for types 1 and 2 [3, 4]. External fixation with HF is recommended as a therapy of choice for OCT type 3 [37]. The possible complications, such as nerve palsies, are explained by pannus creation, which is not primarily associated with HF. Good healing chances are associated with immobilization for 1–3 months [3, 4]. Surgical fusion is indicated only in the case of severe brainstem compression or compression of the upper cervical spinal cord’[22].

### Traumatic injury of C2 vertebra

C2 is the most frequently injured cervical vertebra in children [27]. Successful conservative therapy avoids the limitations on movement reported after surgical fusion [39, 46]. Fulkerson et al. treated two patients with internal fixation and also reviewed similar cases in the literature. They recommended HF as the therapy of choice to prevent possible movement restrictions [16]. They also recommended considering early surgical intervention in cases of odontoid angulation (> 30°), significant displacement, and symptoms of spinal cord injury, due to the higher probability of ligament disruption and the greater instability of the fracture. In line to above opinion recommended Rusin et al. his therapy management [37].

A hangman’s fracture is a hyperextension C2 injury with upper cervical spine fracture, which is almost exclusively seen in children under the age of two. The incidence increases again from the age of 16, where it has the same characteristics as C2 fractures in adults [18, 30, 38, 42]. The recommended treatment for a hangman’s fracture varies according to the type and severity of the fracture instability [2]. Unstable fractures should undergo surgical instrumentation [24, 30, 43]. The main recommendation for traumatic C2 injuries is primary treatment with HF. Cases with angulation or fracture displacement should be treated surgically with internal fixation.

### Complications

Generally, HF was associated with only minor complications including pin loosening, pin tract infection, cranial nerve palsies, and vest-related pressure sores [1, 6, 29, 50]. An anatomical guide for safe pin placement, with recommended pin sizes for different age groups, has been produced by Domenech-Fernandez et al. [13] and Chavasiri et al. [10].

The pinless halo was introduced to reduce the complications associated with HF pins [6]. Only one minor complication (skin irritation) was described in the literature we reviewed [6]. Because of the lack of data in this particular area, more studies are needed to evaluate the use of pinless HF in traumatic instabilities.

## Conclusion

HF seems to play an important role in the treatment of traumatic cervical instabilities in pediatric patients. We suggest that primary therapy with HF is a safe and established procedure. However, there are cases that require treatment with HF in combination with surgery. Most of the studies we found were either retrospective cohort studies or single case series. We did not discover any controlled or randomized studies.

## Limitations

Because of the low incidence of cranio-cervical injuries in children, all studies of HF between 2010 and 2020 provide only limited evidence. No randomized prospective trials on the treatment algorithm for cranio-cervical injuries have been conducted to date. Despite the wide use of HF as the conservative treatment of choice, the lack of clear indication criteria for surgical instrumentation prevents it being considered the standard choice of therapy. Another limitation of this work is that we only searched three databases.

## Data Availability

All data generated or analyzed during this study are included in this published article.

## Abbreviations

AARS: Atlantoaxial rotatory subluxation
AOD: Atlanto-occipital dislocation
C1: First cervical vertebra
C2: Second cervical vertebra
CSI: Cervical spine injury
HF: Halo fixation
OCF: Occipital condyle fracture

## Declarations

### Study design

Systematic review.
